# Multiple primary tumors: a case report and review of the literature

**DOI:** 10.1186/s12891-020-03426-8

**Published:** 2020-06-22

**Authors:** Zhiqing Zhao, Kunkun Sun, Taiqiang Yan, Ran Wei, Wei Guo

**Affiliations:** 1 Musculoskeletal Tumor Center, Peking University People’s Hospital, Beijing, 100044 China; 2 Department of Pathology, Peking University People’s Hospital, Beijing, 100044 China

**Keywords:** Quadruple primary tumors, Gene mutation, Case report, Literature review

## Abstract

**Background:**

Multiple primary tumors, especially quadruple primary neoplasms is extremely rare. Fibrous dysplasia (FD), osteosarcoma (OS), and giant cell tumor of bone (GCTB) are three bone tumors with low incidence while primary pulmonary meningioma is a rare disease. In this case report, we present a unique synchronous occurrence of these four separate pathological conditions.

**Case presentation:**

A 53-year-old male previously underwent resection of OS of fifth rib and FD of eighth rib 1 year ago. Recently, a discontinuous pain at right knee developed. Serial X-ray films showed a progressively pure osteolytic lesion of proximal tibia which extended gradually. The incisional biopsy revealed that this tumor was confirmed as GCTB, and the tumor was successfully managed by extensive curettage and bone cement filling. The diagnosis of GCTB was re-confirmed by the postoperative histopathologic examinations. High-throughput sequencing from the GCTB exhibited a somatic mutation of H3.3A (G35W exon2). Germline testing revealed a germ-cell variant in gene of BRCA2 (exon 8 V220Ifs*4).

**Conclusions:**

This is a unique case with quadruple primary tumors. Germline mutation in gene of BRCA2 may be associated with the occurrence of multiple primary tumors in this patient.

## Background

Multiple primary tumors in the same patient was rarely reported, however, it is not new and already reported in 1921 [1]. Because of recent developments in diagnostic and treatment modalities, reports of four or more primary cancers appear to be increasing. Fibrous dysplasia (FD), osteosarcoma (OS), and giant cell tumor of bone (GCTB) are three bone tumors with low incidence. Primary meningioma occurs in the lung is not common, and only a few cases have been reported since its first report by Kemnitz and Heinrich in 1982 [2–4]. To our knowledge, simultaneous occurrence of these four separate diseases in an individual has not been presented to date. In this case report, we present a unique patient with all the above-mentioned tumors.

## Case presentation

Approval for the study by the local institutional review board was not required because it was a case report. Informed written consent was obtained from this patient.

### Chief complaints

A 53-year-old male who was 170 cm in height and weighed 70 kg (body mass index 24.2) was admitted to our hospital on 6 January 2020, with a chief complaint of pain in the right knee for 7 months. The pain was dull, non-radiating, and intermittent. Symptoms such as fever, fatigue, palpitations, malaise, cough, hemoptysis, breathlessness, and weight loss were absent.

### Medical history

**Fig. 1:**
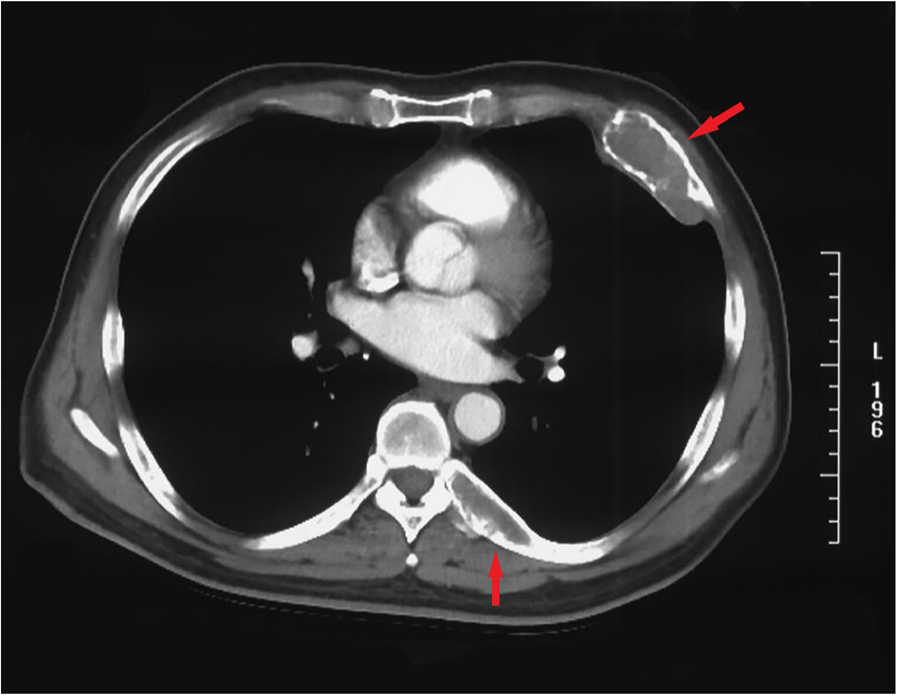
Axial computed tomography image shows the expansive lesion with cortical destruction that originates from the left anterolateral fifth rib, and reveals a well-defined radiolucent unilocular mass that expands the cortical bone of the left posterior eighth rib

**Fig. 2:**
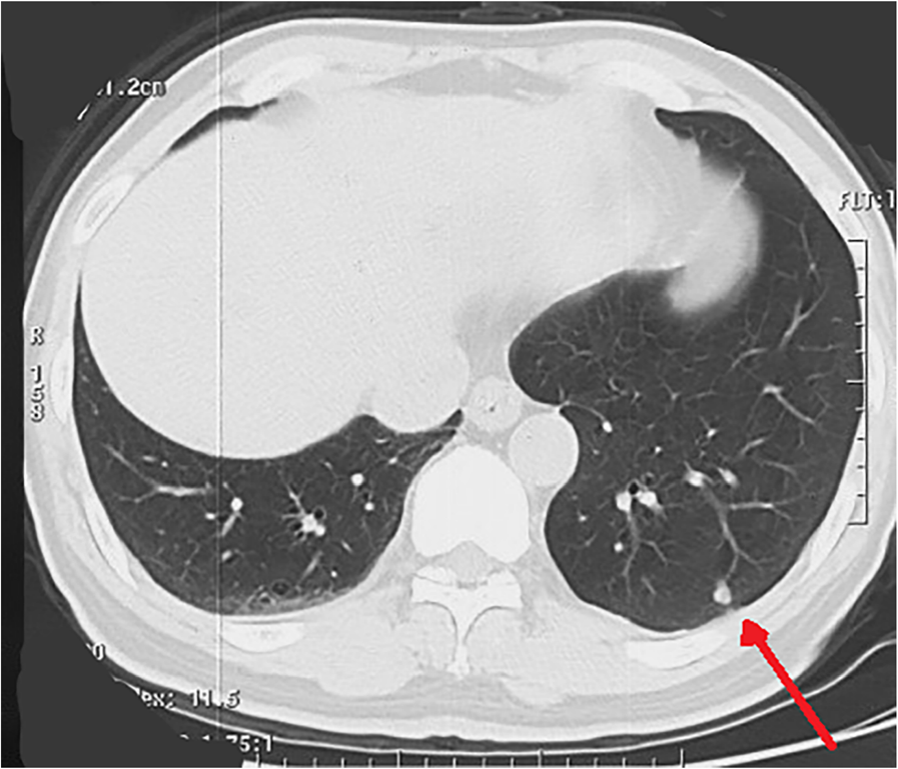
Axial computed tomography image reveals a nodule (0.7 × 0.6 cm) in the left lower lobe (arrowhead)

**Fig. 3:**
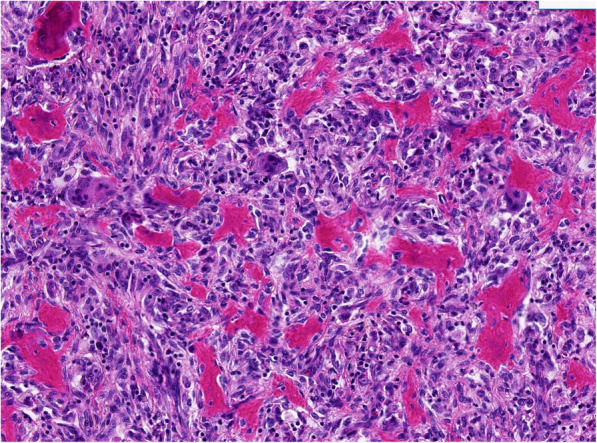
Osteosarcoma. Histopathology shows that spindle cells are minimal atypical and arranged in bundles. Among them, there is irregular bone beam component

**Fig. 4:**
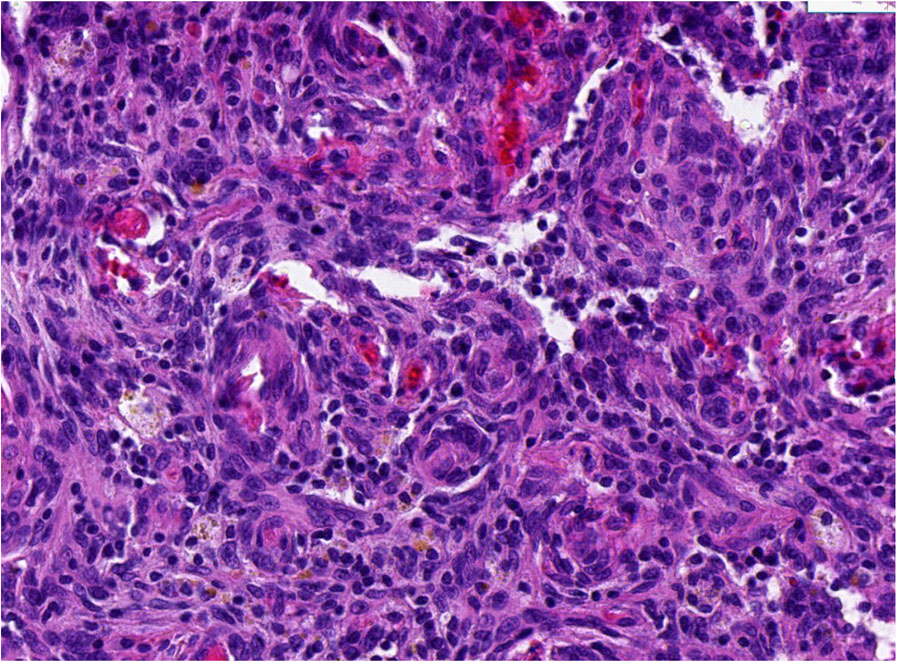
Pulmonary meningioma. Photomicrograph (hematoxylin-eosin stain) shows that the elongated spindle-shaped cells and a delicate fibroconnective tissue are arranged in whorls, or onion peel-like formations

**Fig. 5:**
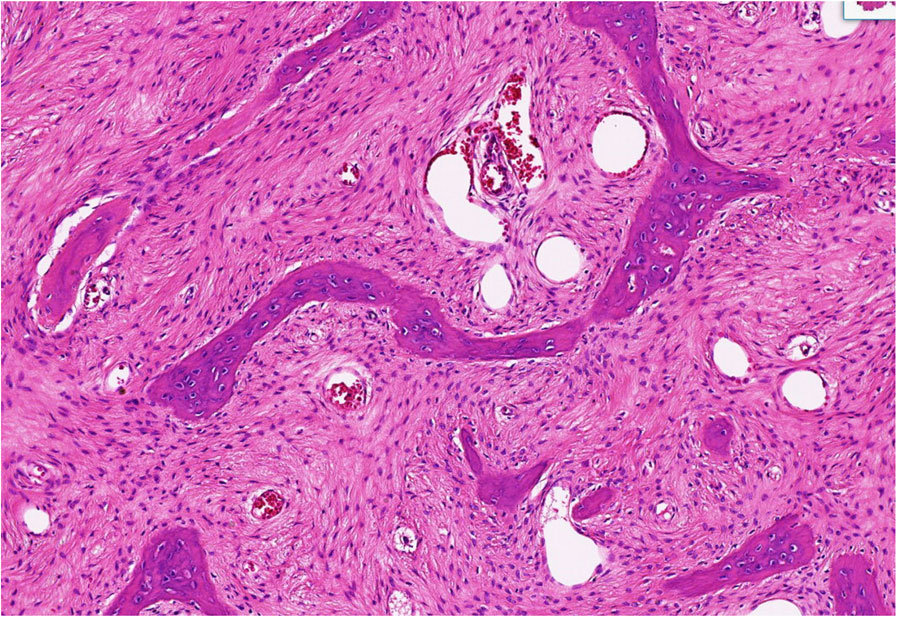
Fibrous dysplasia. Photomicrograph (hematoxylin-eosin stain) shows bland spindle cells and irregular bone trabecula without osteoblasts

The patient denied a history of tuberculosis, diabetes mellitus, hypertension, or coronary heart disease. No specific cancer pedigree was found. He had smoked 20 cigarettes a day for 20 years but did not drink alcohol. The patient has a previous history of triple different neoplasms (treated at an outside hospital) which were as follows: In October 2018, he has intermittent left chest pain and noted a mass at left chest. X-ray film, and contrast enhanced computed tomography (CT) scan of chest revealed a 6.1 × 3.2 cm sized mass in the left fifth rib and a 5.1 × 1.8 cm sized lesion in the left eighth rib, respectively (Fig. 1). Interestingly, the CT scan image also revealed a nodule (0.7 × 0.6 cm) in the left lower lobe (Fig. 2). Cranial CT scan images revealed no abnormality. Subsequent whole-body positron-emission tomography/computed tomography (PET/CT) scan demonstrated expansive destruction of fifth rib (Fig. 3) with increased Fluorine-18-fluorodeoxyglucose uptake (SUVmax: 9.7) and eighth rib (SUVmax: 6.6), and a nodule in the left lower lobe (SUVmax: 2.2). Needle biopsy of fifth rib indicated the diagnosis of OS. Then, the DNA of OS was sequenced alongside with paired constitutional DNA from peripheral blood. The results of OS exhibited mutations in genes of MAP2K1 (K57N, exon-2), H3.3A (G35V, exon-2) and BRCA2 (exon-8 V220Ifs*4). The tumor mutation burden was 0.5 Muts/Mb. Mutation in gene of BRCA2 (exon-8 V220Ifs*4) was confirmed by germline testing. Neoadjuvant therapy was not performed. Thereafter, he received wide tumor resection of left fifth rib and pulmonary wedge resection at November 2018. Specimens from the two different regions were submitted for microscopic examination, respectively. Diagnosis of highly differentiated OS (Fig. 3) of the fifth rib and pulmonary meningioma (Fig. 4) of the nodule of left lower lobe were made. Postoperative chemotherapy was not used due to the type of OS. Four months later, he received tumor resection of left eighth rib. The diagnosis of FD of eighth rib was confirmed pathologically (Fig. 5). All the three diagnoses were confirmed after pathological consultation in different tumor centers. There had been no evidence of recurrence of these tumors at the present consultation.

### Physical examination upon admission

Physical examination of right knee revealed no obvious abnormality.

### Laboratory examinations

His serum tumor markers and other laboratory values were all within normal range.

### Imaging examinations and biopsy

**Fig. 6:**
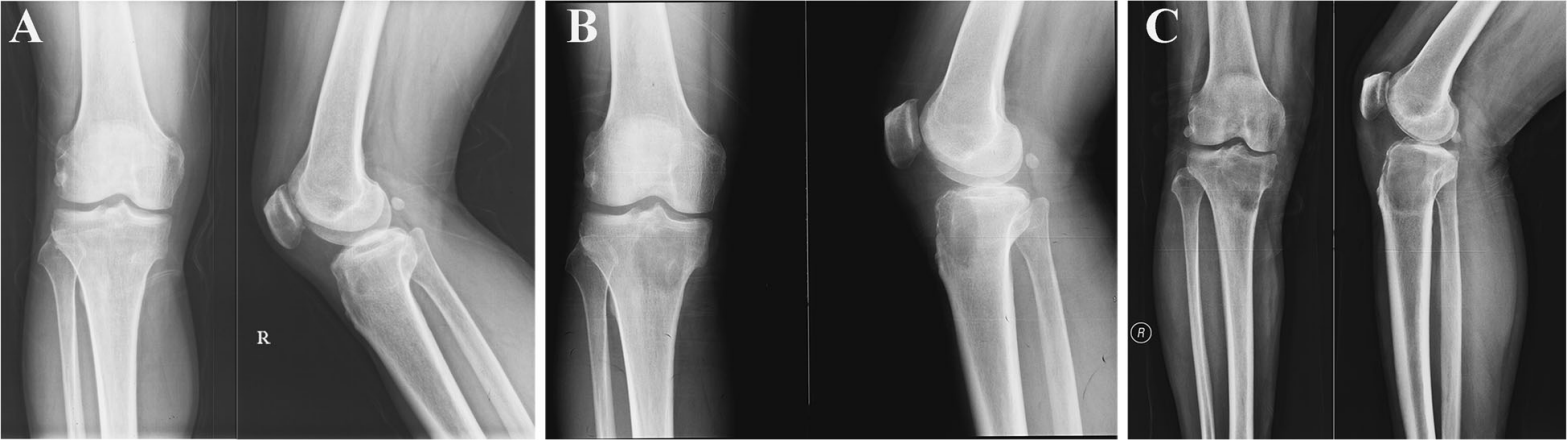
**a** Anteroposterior radiograph of the right knee showed a small lytic lesion in the proximal tibia with a diameter of 1 cm. **b** After five months, the lesion extended, and has a non-sclerotic margin medially (arrowheads). **c** After six months, the lesion shows typical appearance of GCTB which extends to the subchondral bone that is eccentric in location and extends to the subchondral bone with a sclerotic margin

**Fig. 7:**
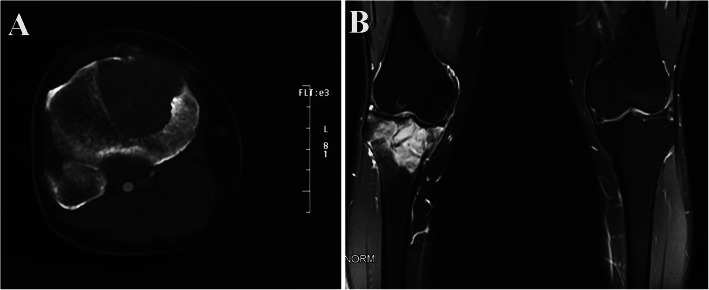
CT scan (**a**) and MRI (**b**) of right tibia showed a lytic lesion in the proximal tibia without presence of soft-tissue mass

From May 2019 to January 2020, serial X-ray films showed a pure osteolytic lesion at the right proximal tibia which extended gradually (Fig. 6). The latest CT scan images and magnetic resonance imaging (MRI) also revealed a mass of 4.4 × 4.0 cm located in the right proximal tibia with an indistinct boundary (Fig. 7). Bone scintigraphic imaging depicted a high uptake at the right proximal tibia. Subsequently, incisional biopsy of the tibia lesion was performed and a histopathological diagnosis of GCTB was made. Also, this diagnosis was confirmed by four different pathology departments.

### Final diagnosis

The patient was diagnosed with multiple primary neoplasms including GCTB of tibia, OS of fifth rib, FD of eighth rib, and pulmonary meningioma.

### Treatment and postoperative pathological findings

**Fig. 8:**
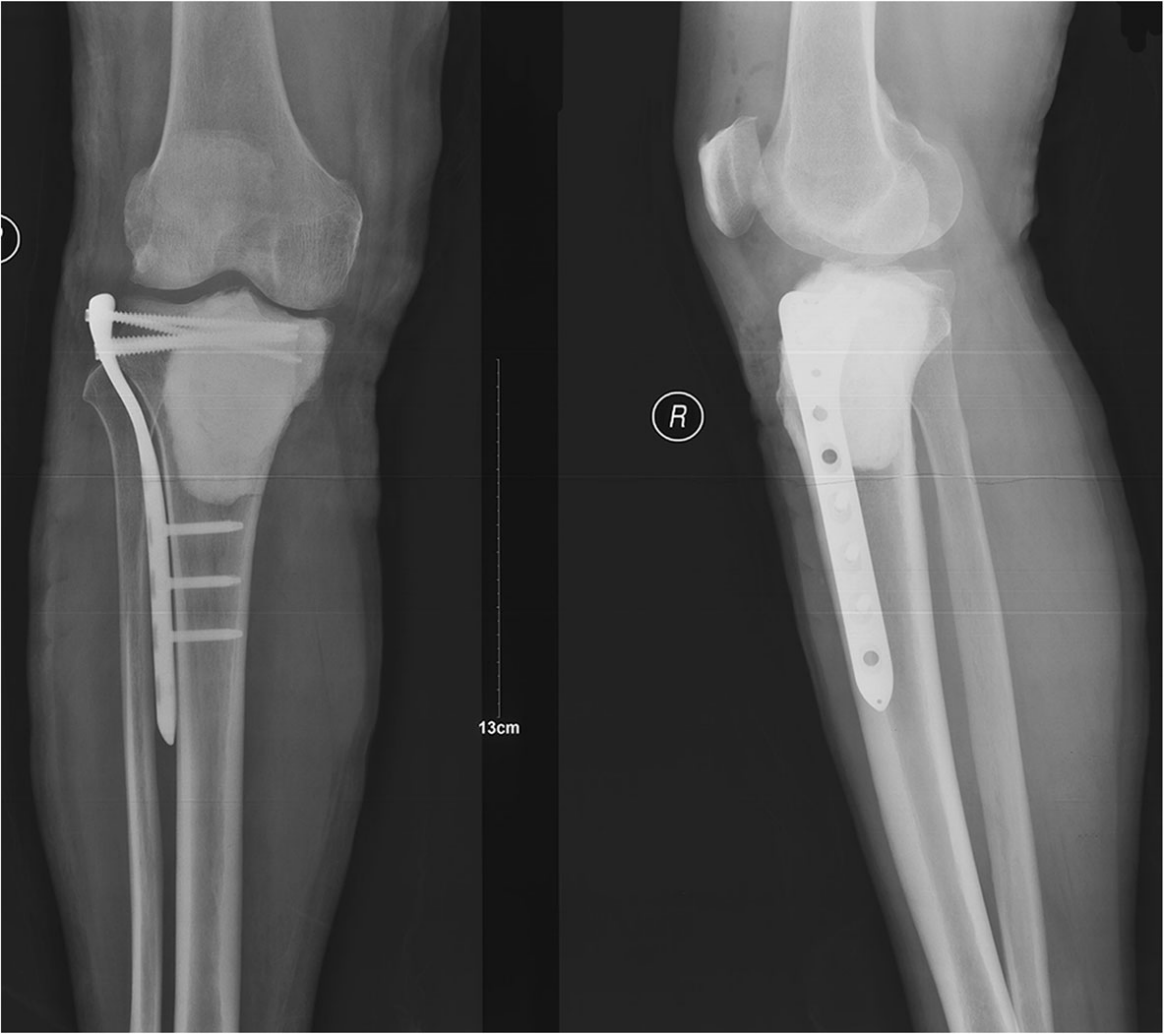
The postoperative X-ray film of the right tibia

**Fig. 9:**
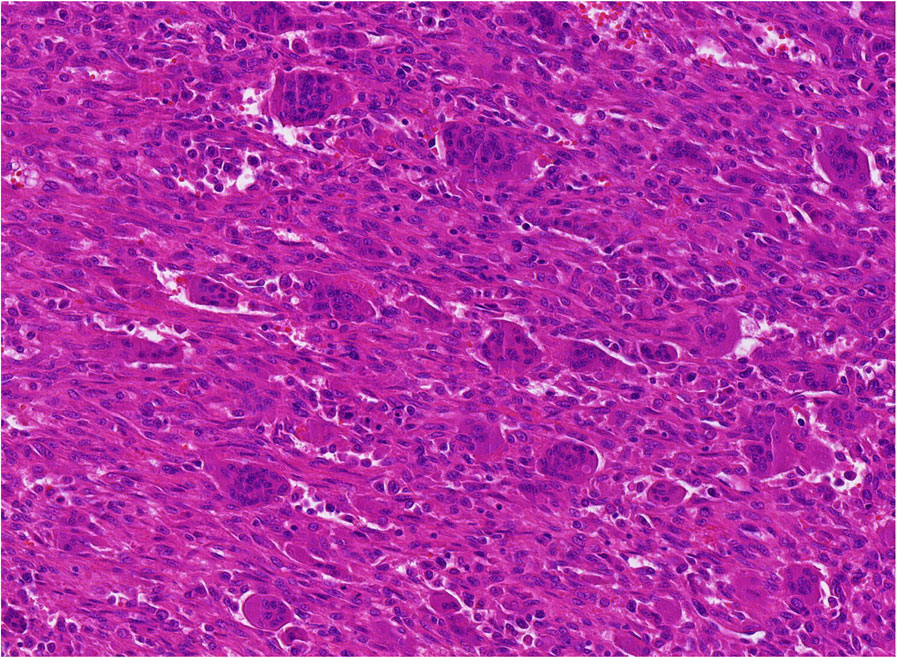
Giant cell tumor of bone. Photomicrograph (hematoxylin-eosin stain) shows a background of monoclonal stromal cells and the presence of multinucleated giant cells

**Fig. 10:**
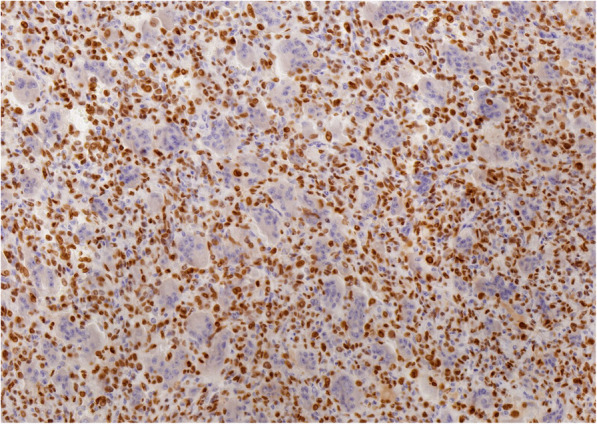
Immunohistochemical staining of tumor cells is strongly positive for H3.3A(G35W), indicating GCTB

On 7 January 2020, the patient provided a written informed consent to and underwent curettage of the tumor, filling of bone cement and plate fixation under general anesthesia (Fig. 8). Intraoperative photograph shows a destruction of the proximal of the lateral tibia without soft-tissue mass. Tumor mass was confined within the cortex of tibia. Resected specimen from the proximal tibia were submitted for microscopic examination and a diagnosis of GCTB was confirmed (Fig. 9). Immunohistochemical findings were as follows: CD68 (+); CD163 (+); P53 (+,5%); LCA (−); Ki-67 (+, 10%); and SATB2(+); H3.3A(G35W)(+); PD-L1-Ventana (CPS +) (Fig. 10). After written informed consent had been obtained, high-throughput sequencing of DNA from the tumor of tibia and peripheral blood (lymphocytes) was implemented. Testing from the GCTB revealed mutations in genes of H3.3A (G35W exon-2) and BRCA2 (exon-8 V220Ifs*4), and the tumor mutation burden was 0.5 Muts/Mb. Germline testing confirmed a pathogenic variant in gene of BRCA2 (exon-8 V220Ifs*4). No other family members were tested because they lived in distant areas. The patient recovered smoothly. And he could walk without crutch 1 month after surgery. There was no sign of local recurrence of the ribs and tibia at the latest followup (5 months after surgery).

## Discussion

The multiple primary tumors are now detected increasing due to prolonged survival time of cancer patients and developments in diagnostic techniques. However, the occurrence of quadruple cancer still extremely low. Including autopsy cases, the reported incidence of quadruple cancer is 0.007% [5, 6]. In this case report, we described a synchronous occurrence of four separate pathological conditions, and discussed the management of multiple primaries.

Multiple primary tumors are defined as more than one synchronous (within 6 months) or metachronous tumors in the same individual. The definitions of multiple primary tumors differ from one study to another. It can be confirmed if tumors arise in different sites and/or are of a different histology. Besides, it should be rule out the possibility of one lesion is a metastasis of others, or second cancers [7]. Longhi et al. [8] have found that second cancers are treatment-related disease, which occur 3.6% of bone sarcoma survivors within 10 years. The most common second cancers include breast carcinoma, leukemia, sarcomas, and salivary gland neoplasms. In our study, we defined this “multiple primary tumors”, consisting of benign tumor and malignant tumor rather than all malignances, which was not identical to previous studies. The present case met the criteria that each tumor is distinct with different histopathology, and diagnosed to have asynchronous quadruple tumors.

A literature review of quadruple primary neoplasms was performed after searching the PubMed database. Only 15 cases of clinical quadruple primary neoplasms have been reported between 2000 and 2020. Table 1 shows a summary of the previous studies describing multiple primaries. We can see that sequencing of tumor genomes was performed in only two cases [14, 16]. Furthermore, we are aware of that there has no report of patient with quadruple neoplasms which consist of three bone tumors and a primary pulmonary meningioma.

**Table 1:** The cases of quadruple primary neoplasms reported in the English literature between 2000 and 2020

Author, year	Age/Gender	Tumors	Type	Survival	Genetic test
Mussari [9], 2000	47/M	Anus/ Esophagus/ Lung/ Oral cavity	N/A	N/A	N/A
	48/M	Lung/ Stomach/ Rectum/ Lung	N/A	N/A	N/A
	55/M	Oral cavity/ Oropharynx/ Esophagus/ Melanoma	N/A	N/A	N/A
	60/M	Oral cavity/ Larynx/ Lymphoma/ Esophagus	N/A	N/A	N/A
Noh [10], 2008	68/F	Breast cancer/ Rectal adenocarcinoma/ Ovary carcinoma/ Endometrium carcinoma	Metachronous	N/A	N/A
Angurana [11], 2010	35/F	Infiltrating duct cell carcinoma Breast carcinoma/ Early infiltrating duct cell carcinoma of breast carcinoma/ Adenocarcinoma endometrium/ Esophagus carcinoma	Metachronous	30 years	N/A
Jiao [12], 2013	64/M	Small intestine adenocarcinoma/ Colon adenocarcinoma/ Urothelial carcinoma of the pelvis/ Pancreatic cancer	Metachronous	20 years	N/A
KIM [13], 2013	73/F	Thyroid carcinoma/ Breast carcinoma/ Pancreatic carcinoma/ Gastrointestinal stromal tumor	Synchronous	8 months (DOD)	N/A
Milosevic [14], 2014	40/F	Medullary thyroid carcinoma/ Micropapillary thyroid carcinoma/ Melanoma/ Breast carcinoma	Metachronous	N/A	PTEN/P53 /RET/HRAS/KRAS
Kousaka [15], 2014	41/F	Osteosarcoma/ Tongue cancer/ Thyroid cancer/ Breast carcinoma	Metachronous	3 months	N/A
Grace [16], 2015	70/M	Glioblastoma/ Schwannoma/ Neuroendocrine tumor/ Adenoma	Synchronous	N/A	EGFR /CDKN2A/2B/PTEN
Meeks [17], 2016	95/F	Adenocarcinoma/ Adenoma/ Neuroendocrine tumor/ Schwann cell hamartoma	Synchronous	N/A	N/A
Elec [18], 2017	78/M	Prostate adenocarcinoma/ Clear-cell renal carcinoma/ Papillary renal carcinoma/ Bladder cancer	Synchronous	4 years	N/A
Nanashima [19], 2017	67/M	Stomach/ Sigmoid colon/ Rectum/ Pancreas carcinomas	Synchronous	4.25 years	N/A
Wang [20], 2019	56/F	Cervix/ Endometrium/ Ovary/ Stomach carcinomas	Synchronous	1 year	N/A

*M* male, *F* female, *N/A* not applicable, *DOD* died of disease

OS is the most common primary malignant tumor, which generally arise around the knee. OS that occurs primarily in rib is very rare [21]. Burt et al. [22] have reported that only 13 (0.9%) of 1435 OS cases arose from ribs. The main treatment for OS is tumor resection with or without chemotherapy. Primary meningioma in lung is usually a benign disorder, presenting as a solitary and slow growth nodule. It has a satisfactory prognosis after surgical resection [23]. FD is a benign skeletal disorder and can affect any bones. It can develop malignant transformation with a reported rate ranging from 0.4 to 6.7% [24]. In the present case, the FD of rib and pulmonary meningioma were asymptomatic and were diagnosed incidentally. And all lesions were resected successfully. GCTB is a locally aggressive primary bone tumor that is characterized by mononuclear stromal cells and osteoclast-like giant cells, and it usually occur in the subarticular (epiphyseal/epimetaphyseal) location. The main treatment of GCTB was surgical curettage with cement (polymethylmethacrylate) placement, with recurrence rate of 15 to 25%. Regarding the lesion of proximal tibia, biopsy has proved the diagnosis of GCTB rather than metastasis of OS. Therefore, we performed the traditional treatment of curettage and filling of cement.

The reasons of quadruple neoplasms are unclear, which may be genetic mutation, environmental factors, smoking, radiation exposure, etc. [5, 7, 25]. Review the literature, many of gene mutations have been implicated in the pathogenesis of neoplasms. As is well-known, the Cowden syndrome may be caused by transgenation of PTEN [26, 27]. A woman presenting with mutation of CHEK2 gene has a high risk of suffering breast and thyroid carcinomas [28]. Germline mutations in the CDKN2A (p16) gene may result in cutaneous melanoma [29–31].

It is important to establish whether these tumors are hereditary or not by interviewing patients with multiple primary neoplasms. This information might be helpful for doctors to assess cancer risk and to optimize treatment. In this present case, however, the family history is negative. And, he has no history of chemotherapy or radiation exposure. The patient’s heavy smoking may be a possible cause. To further find the genetic reason, we performed the high-throughput sequencing to test the DNA of tumor tissue and peripheral blood. In expectation, we found the germline mutation in gene of BRCA2. Meanwhile, the somatic mutations in MAP2K1 (K57N, exon-2) and H3.3A (G35V, exon-2) were detected in OS and mutation in H3.3 (G35W exon2) in GCTB, respectively.

As an autosomal dominant tumor suppressor gene, the gene of BRCA2 or BRCA1 contribute to the repair of DNA. A great number of studies have analyzed the relationship of BRCA genes and cancer susceptibility [32, 33]. Prior studies have illustrated that mutation in BRCA2 would increase the risk of breast cancer, ovarian carcinoma, prostate cancer and other tumors. In a recent study by Kovac et al. [34], mutations in BRCA2 or BRCA1 genes was a cause of occurrence of OS. Although the bone tumors associated with mutations in BRCA2 have been seldom studied, and the possible function of BRCA2 is far less certain, we think the germ-cell mutation of BRCA2 may be the main reason of occurrence of quadruple neoplasms for this case. The product of BRCA2 regulates transcription, while some mutations in BRCA2 would change the process. Recent word has concluded that if the gene of BRCA2 overexpressed, p53 transcriptional activity would be down-regulated [35]. It is unclear how the mutation of BRCA2 in this patient result these four tumors. Further study is required to clarify the function of BRCA2.

Many studies have reported 95% of GCTB with the H3.3A mutation, and the majority of them are represented (G35W) [36–38]. In this patient, sequencing of DNA from the tumor of tibia showed a SNV mutation of H3.3A (G35W exon-2), which is in agree with the reported outcome. Meanwhile, the mutation of H3.3A verified the diagnosis of GCTB of tibia.

This case report has a limitation that we did not test for the specific mutation of the patient’s relatives.

In conclusion, molecular testing can provide insight into the diagnosis, treatment, and underlying etiology of the tumors. This case also potentially expands the constellation of neoplasms associated with germline BRCA2 mutation. We believe that this is a useful addition to the literature.

## Data Availability

The datasets used and/or analyzed during the current study are available from the corresponding author on reasonable request.

## Abbreviations

FD: Fibrous dysplasia
OS: Osteosarcoma
GCTB: Giant cell tumor of bone
CT: Computed tomography
PET/CT: Positron-emission tomography/computed tomography
MRI: Magnetic resonance imaging
